# Do we still need supertrees?

**DOI:** 10.1186/1741-7007-10-13

**Published:** 2012-02-27

**Authors:** Arndt von Haeseler

**Affiliations:** 1 Center for Integrative Bioinformatics Vienna, Max F Perutz Laboratories, University of Vienna and Medical University Vienna, Dr.-Bohr-Gasse 9, 1030 Vienna, Austria

## Abstract

The up-dated species level phylogeny for the carnivores using a supertree approach provides new insights into the evolutionary origin and relationships of carnivores. While the gain in biological knowledge is substantial, the supertree approach is not undisputed. I discuss the principles of supertree methods and the competitor supermatrix approaches. I argue that both methods are important to infer phylogenetic relationships.

See research article http://www.biomedcentral.com/1741-7007/10/12

## 

After more than two centuries of intensive research the phylogeny of such important groups as the carnivores, let alone deeper phylogenetic relationships, is still not satisfactorily settled. Recently, Nyakatura and Bininda-Emonds [1] published an up-dated phylogeny of all 286 carnivore species. The new phylogeny is an extension of a study 12 years ago [2], which needed revision due to better methodologies and more data, especially DNA sequence data.

However, the reconstruction of the carnivore tree is not as straightforward as one may expect. Typically, DNA or protein sequence data or phylogenies are available for only a subset of the carnivores. Thus, the major challenge is to construct one phylogeny for a taxonomic group from multiple sources. To this end the authors analyzed 241 trees available from the literature and additionally 74 gene trees generated from sequence data. The total of 341 so-called source trees was then combined into one supertree, assumed to mirror the phylogeny of the carnivore species. Combining trees derived from different data sets falls into the realm of supertree methods [3]. Alternatively one may also apply the so-called supermatrix method to combine data [4]. Here, the character data are pooled and then followed by a tree reconstruction. Both methods are in wide use and it is still an open question which method is preferable.

## Building supertrees using matrix representations with parsimony: a robust approach?

**Figure 1:**
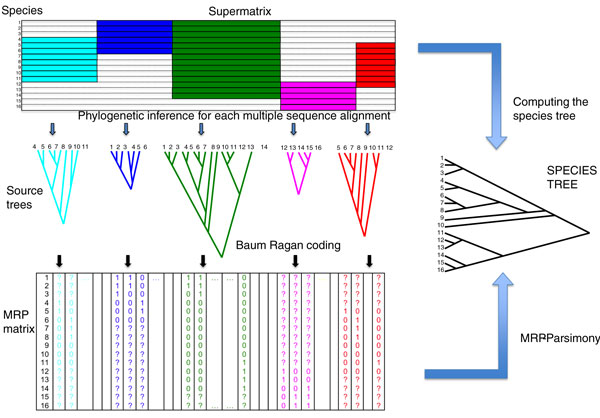
**A comprehensive workflow for supertree and supermatrix approaches applied to DNA data**. In the supertree approach, phylogenetic trees are reconstructed for each of the five genes. The resulting source trees are recoded using the Baum Ragan coding to obtain the MRP matrix. Based on this matrix the species tree is computed using MRP parsimony. The supermatrix approach takes the concatenated gene alignments and computes the species tree directly using standard tree reconstruction methods.

Supertree methods combine source-trees, or trees obtained from the literature, with overlapping species sets into one tree. Nyakatura and Bininda-Emonds [1] selected matrix representation with parsimony (MRP) [5] as the method of choice for generating a supertree of carnivore species. The workflow is illustrated in Figure 1: MRP constructs a new data matrix (MRP-matrix), where each species in the source-trees is represented in a row. The columns of the MRP-matrix are built by encoding the source-trees. Species sharing a common node in the rooted source-tree are assigned the character '1'; the remaining species in the tree receive character '0'. Species not in the source-tree are assigned the character '?'. Thus, each branch of each source-tree contributes one column to the matrix representation of the data. The MRP-matrix resembles a multiple sequence alignment with binary characters {0,1} and missing characters {?}. This superficial 'homology' is employed to reconstruct the most parsimonious tree(s) of the encoded branches from the source trees [3]. The supertree (or supertrees) displays the phylogenetic relationship of all species in the source trees. However, contrary to multiple sequence alignments obtained from DNA or proteins, the variability we observe in the MRP-matrix cannot be modeled by probabilistic models of evolutionary change.

Like almost all phylogenetic methods that deal with large collections of heterogeneous data, many pitfalls during compilation and analysis of the data exist (reviewed in [6]). Nyakatura and Bininda-Emonds [1] did a great deal of work to avoid such systematic errors. Critical issues are the quality of the source trees, the fact that different source trees may have used overlapping raw data and are thus no longer independent, and that it is not at all obvious how to weight the source trees.

With the increasing availability of molecular sequence data, this classical method of supertree reconstruction will soon be replaced by supertree analysis on molecular data, which avoids potential dependency problems. All that will be required is simply to derive a tree for each multiple sequence alignment and apply MRP to the inferred source-trees. In such situations, the tedious compilation of source trees from the literature is not necessary. The carnivore supertree in [1] includes already 74 gene-trees. On the other hand, including source trees from the literature provides phylogenetic information for species for which no molecular data are yet available, as is the case for 57 out of 286 carnivore species.

Finally, contrary to modern phylogenetic inference, the supertree approach lacks any statistical model of evolutionary change, although supertree methods still infer the 'true' relationships very well. Thus, the phylogenetic information presented in source trees together with a careful analysis is able to reconstruct the phylogeny of large systematic groups. Some progress has been made to include statistical analysis into a supertree approach. For example, bootstrap methods were applied to evaluate the support for the supertree by randomly sampling with replacement from the source-trees. Recently, a new approach for supertree reconstruction was proposed: matrix representation with likelihood (MRL) [7]. MRL is one approach towards more statistical thinking in supertree reconstruction.

## Supermatrices: concatenation of raw data into super alignments

Supermatrix methods work directly on the raw data that are used for phylogenetic analysis - for example, multiple sequence alignments of DNA sequence (as shown in Figure 1). This method is sometimes referred to as 'concatenation' or 'total evidence' and the multiple sequence alignments used are referred to as 'systematic characters'. Groups of these are concatenated into one supermatrix. In this era of phylogenomic analyses, supermatrix methods are widely used. We will only discuss supermatrix methods that use sequence alignments as the source for the tree reconstruction. Other methods exist that combine molecular data with morphological data to reveal additional phylogenetic signals, but it is then not clear how to reconstruct the tree using generally accepted models of evolution. Modeling the evolutionary process of DNA (or amino acid) sequences is well understood and thus the entire repertoire of model-based phylogenetic analyses is at hand to infer a phylogeny from a supermatrix that is constructed from different multiple sequence alignments (Figure 1). Because it is claimed that supermatrix approaches use the phylogenetic information encoded in the characters more fully than supertree methods [4], supermatrix approaches seem to be superior. Thus, it is not surprising that supermatrix methods are *en vogue *and one gets the impression that they are beginning to replace supertree methods. Nyakatura and Bininda-Emonds [1] realized this and inferred a carnivore phylogeny from a concatenated sequence alignment (44,000 base pairs for 229 carnivores). Surprisingly, the resulting branching pattern did not deviate dramatically from the supertree. However, some differences were detected and it will be important to explore and discuss these further in order to ultimately resolve the phylogenetic relationships of the carnivores - and also to understand the limits of supermatrix and supertree approaches.

Supermatrix methods also have potential pitfalls that are not so different from those affecting supertree methods. Almost all phylogenetic tools treat the characters in the supermatrix as independent. This is not true for most sequences and therefore may lead to systematic errors. Another potential pitfall is that although tree reconstruction methods include very complex models of sequence evolution, they cannot yet account for the complexity in superalignments. Finally, the assumption that gene trees are identical to speciation trees is not necessarily true and this introduces another potential bias [8].

One obvious caveat for supermatrix and supertree approaches, if they deal with molecular data, is that we have to ensure that DNA sequences included in the alignment are orthologous: that is, two genes from two species evolved from a single gene in the last common ancestor of both species [9]. If the orthology assumption does not hold, then both approaches will produce misleading trees.

## Supermatrix methods complement, but cannot yet replace, supertree methods

At first glance it may appear that supermatrix methods are superior to supertree methods; however, both methods make a series of simplifying assumptions at different stages of data compilation and data processing that makes it difficult to judge which, if either, is actually superior. Supermatrix methods have the charm of being amenable to statistical analysis, something that is currently underdeveloped in supertree methods, but even a statistically significant result can be wrong if systematic errors are not eliminated.

One way to understand the impact of simplifying assumptions on the resulting tree is to use simulations, where the truth is known ([10] and references therein). Unfortunately, simulations can cover only a tiny, tiny fraction of the universe of possible evolutionary scenarios. Thus, they only allow us to exclude phylogeny reconstruction approaches that already fail to show good performance for the selected simulation conditions. However, the converse is not true. The good performers under selected conditions are not necessarily good performers under all conditions. That is why all simulations have only a limited explanatory power. While the study by Kupczok and colleagues [10] shows that supermatrix methods usually have a higher probability of inferring the truth, MRP-supertree methods are runners-up and are superior to supermatrix approaches in the case of significant disagreement between gene trees and the species tree. One should also note that the simulations refer to a sequence-based approach. It is at present unclear how to include morphological characters, due to the lack of generally accepted models. Thus, supermatrix approaches may be favored in the simulations.

Thus, for the time being, whenever one wants to study the evolutionary relationships of living organisms it is possibly best to apply many reconstruction methods and to discuss the differences and commonalities of the resulting trees. Only then one can distinguish between reconstruction artifact and true evolutionary history. Nyakatura and Bininda-Emonds [1] discuss the outcome of different phylogeny reconstruction methods to avoid inferring wrong phylogenetic relationships. The ever increasing production of new sequence data and our increased ability to deal with complex models of sequence evolution will certainly lead to a further revision of the carnivore tree in ten years. However, the phylogeny presented today will help to understand where information is missing that needs to be filled in during the coming years.

Scientific progress draws upon the application of different methodologies to the same problem. Only conflicts in the results will lead to progress in our understanding of phylogenies and the relationship among organisms. We understand a lot about how phylogenetic inferences work, but our understanding of how tree inference from patchy data works is still in its infancy. The simulation studies published are too simplistic to come to sound conclusions. Thus, supertree methods carefully applied are still valid and relevant for phylogenetic inference.
